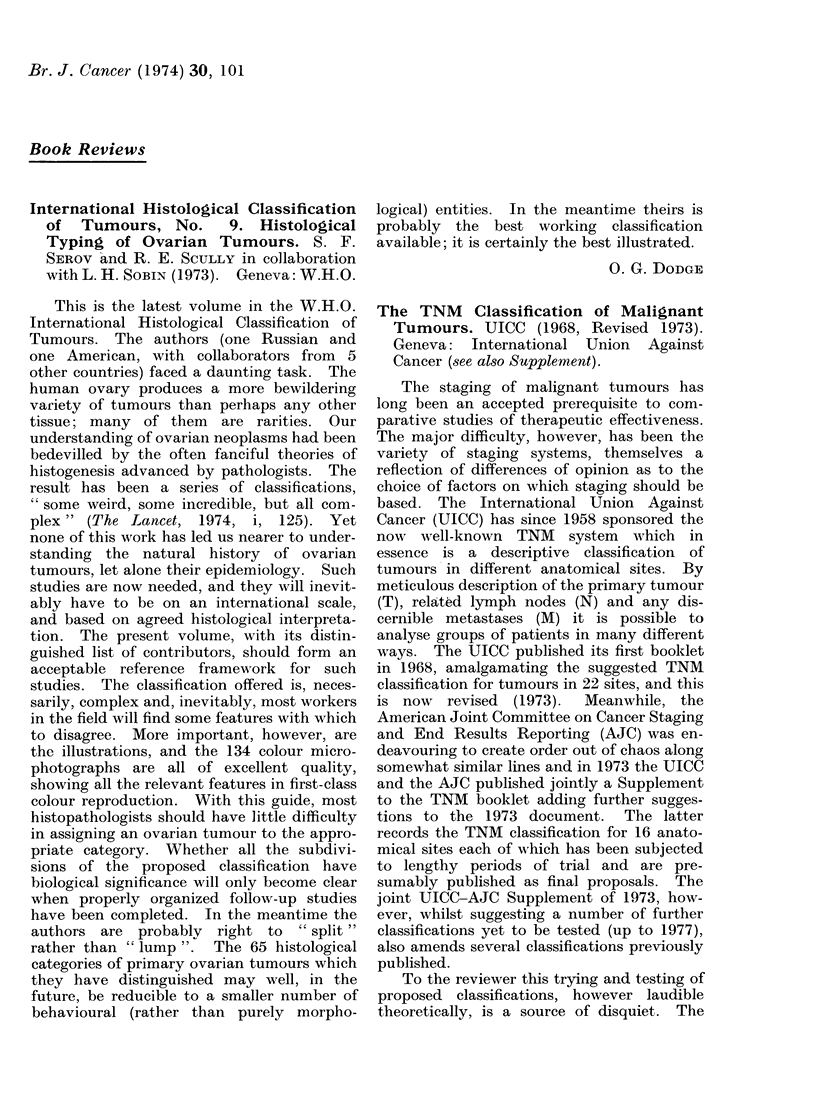# International Histological Classification of Tumours, No. 9. Histological Typing of Ovarian Tumours

**Published:** 1974-07

**Authors:** O. G. Dodge


*Br. J. Cancer* (1974) **30**, 101

### *Book Reviews*

**International Histological Classification of Tumours, No. 9. Histological Typing of Ovarian Tumours.** S. F. SEROV and R. E. SCULLY in collaboration with L. H. SOBIN (1973). Geneva: W.H.O.

This is the latest volume in the W.H.O. International Histological Classification of Tumours. The authors (one Russian and one American, with collaborators from 5 other countries) faced a daunting task. The human ovary produces a more bewildering variety of tumours than perhaps any other tissue; many of them are rarities. Our understanding of ovarian neoplasms had been bedevilled by the often fanciful theories of histogenesis advanced by pathologists. The result has been a series of classifications, " some weird, some incredible, but all complex " (*The Lancet*, 1974, i, 125). Yet none of this work has led us nearer to understanding the natural history of ovarian tumours, let alone their epidemiology. Such studies are now needed, and they will inevitably have to be on an international scale, and based on agreed histological interpretation. The present volume, with its distinguished list of contributors, should form an acceptable reference framework for such studies. The classification offered is, necessarily, complex and, inevitably, most workers in the field will find some features with which to disagree. More important, however, are the illustrations, and the 134 colour microphotographs are all of excellent quality, showing all the relevant features in first-class colour reproduction. With this guide, most histopathologists should have little difficulty in assigning an ovarian tumour to the appropriate category. Whether all the subdivisions of the proposed classification have biological significance will only become clear when properly organized follow-up studies have been completed. In the meantime the authors are probably right to " split " rather than " lump ". The 65 histological categories of primary ovarian tumours which they have distinguished may well, in the future, be reducible to a smaller number of behavioural (rather than purely morphological) entities. In the meantime theirs is probably the best working classification available; it is certainly the best illustrated.

O. G. DODGE